# Epstein barr virus IgG and EBER-1 in Burkitt’s lymphoma children at a referral hospital in western Kenya

**DOI:** 10.11604/pamj.2019.34.206.20244

**Published:** 2019-12-19

**Authors:** Isaac Ndede, Simeon Kipkoech Mining, Kirtika Patel, Fredrick Muyoma Wanjala, Constance Nalianya Tenge

**Affiliations:** 1 Department of Immunology, Moi University School of Medicine, Eldoret, Kenya; 2 Department of Biological Sciences, University of Eldoret, Eldoret, Kenya; 3 Department of Child Health and Paediatrics, Moi University School of Medicine, Eldoret, Kenya

**Keywords:** Burkitt lymphoma, non-Hodgkin lymphoma, EBV IgG, EBER-1

## Abstract

Burkitt's lymphoma (BL) is a frequent childhood B cell non-Hodgkin's lymphoma (NHL) in equatorial Africa associated with infections. Chronic Epstein Barr virus (EBV) infections can lead to host immune stimulation that may trigger genetic translocation(s), neoplastic transformation and proliferation of B cells. We determined EBV immunoglobulin G (IgG) in sera from participants and EBER-1 in tumour sections in confirmed BL cases at Moi Teaching and Referral Hospital (MTRH). A cross sectional study of children with clinical and histology diagnosis of NHL from whom BL status were confirmed by immunohistochemistry (IHC) was carried out. Epstein Barr virus IgG in sera was determine using Enzyme-linked immunosorbant assay, IHC for EBER-1 and MYC protein in tumour sections. Demographic and clinical information were obtained from questionnaires and hospital files respectively. Ninety three percent of sera were EBV IgG positive of which 31.7% were confirmed as BL. All jaw BL tumours and 86.7% of BL tumours carried EBER-1 antigen. Odds ratio of EBER-1 positive was 1.39, 95% CI: 0.16-12.19 in BL tumours regardless of age or gender. EBV infection among the study participants may be associated with BL, however, EBER-1 and MYC negative in BL tumours suggest alternative BL pathogenesis or variant.

## Introduction

Burkitt's lymphoma (BL) is a tumour of the lymphoid tissues of three different variants; endemic (eBL), sporadic (sBL) and immunodeficiency related. The endemic type has been is common in equatorial Africa. Burkitt's lymphoma has a non-random clustering and overlap of with ecological zone of human pathogens such as Plasmodium falciparum, EBV, Kaposi's sarcoma herpes virus (KSHV), human herpes virus 8 (HHV-8) and human immunodeficiency virus (HIV) in this region [1,2]. Endemic BL commonly involve certain anatomic sites; the jaws tend to predominate in younger children while abdominal locations occur more frequently with increasing age. Sporadic BL occurs worldwide with no geographic or climate association and no apparent infection in its aetiology. This variant usually presents as intra-abdominal swelling and rarely involves the jaw in young adults [3]. Epstein Barr virus is known to preferentially infect resting and memory B lymphocytes. During infection, virus binding is mediated by envelope glycoproteins gp 350 and gp 42 to complement receptors 2 (CR2) and HLA class II proteins on target cells respectively. Majority of EBV infected B cells are recognized and eliminated by host T cell-mediated immune responses, but a fraction of the virus usually remain and enter latency I phase within the germinal centre where they express only three viral genes [4], characterized by expressions of small non coding EBV RNAs (EBERs) and EBNA1.

Latency II is characterized by expression of EBNA-1, latent membrane protein-1 (LMP-1), LMP-2, EBERs and is associated with Hodgkin's lymphomas. While latency III viral products include all EBNAs, EBERs, LMPs, non-translated RNA's-microRNA (miRNA) and is frequently observed in post-transplant lymphoproliferative disorders [5]. Some EBV genome products exhibit homology to a wide variety of cell molecules, such as cytokines and signal transducers important in promoting humans infectivity. Epstein Barr virus proteins such as LMP-1 and LMP-2 have growth transforming ability and may allow unchecked cellular proliferation lymphoma pathogenesis. Another product BHRF-l, a homologue of BCL-2 family, is a known apoptosis suppressor. Growth transformation and uncontrolled proliferation are thought to facilitate oncogenic translocation during pre-B-cell development and immunoglobulin rearrangement [6]. Immune stimulation in response to persistent infections such as EBV in a population probably increases the risk of oncogenic mutations and neoplastic transformation. In addition, climatic and ethnogeographic factors such as, exposure to ultraviolet light, chemical carcinogens, genetic factors and cultural practices among populations have been reported to influence temporal and/or spatial clustering of lymphoma including BL [7]. Our study sought to determine EBV IgG in serum, EBER-1 and MYC in tumours sections in children and adolescent diagnosed with BL at Moi Teaching and Referral Hospital (MTRH) in Eldoret, Kenya.

## Methods

A cross sectional study of children and adolescents ≤18 years old with clinical and histology diagnosis of non-Hodgkin's lymphomas, were targeted at MTRH in Eldoret, Kenya, before onset of treatment. Blood and biopsy samples were taken from each patient whose parent or legal guardian provided written informed consent, in accordance with Institutional Research Ethics Committee (IREC). Participants' files and questionnaires were used to obtain clinical and demographic information. To determine EBV IgG, patient sera and controls were incubated in microtitre strip wells coated with EBV-capsid antigen using Enyzme-linked immunosorbent assay (ELISA) kit (Human Gesellschaft, Germany), the plates were then washed and absorbance measured at 450nm within 30 minutes of terminating the reaction in an ELISA microplate reader. Patient's value equal or greater than cut-of-values ± 15% were considered EBV-IgG-antibody positive. Three (3) μm tissue sections from immunohistochemistry confirmed BL tumours (results not shown), were deparaffinised in xylene and rehydrated in a graded series of ethanol before being subjected to epitope retrieval prior to staining with EBER-1 and MYC (DAKO^®^) primary antibodies, interposed with washing steps using tris-buffered saline (TBS) at pH 7.6. Endogenous peroxidise was neutralised by peroxidise followed by protein block to reduce non-specific binding of EBER-1 antibody. Followed by 3, 3'-diaminobenzidine tretrahydrochloride dehydrate (DAB) to visualize antibody binding after incubation at room temperature in a Leica Bond III® stainer. Staining relativities were evaluated in at least 10 high-power microscopic fields of the slides. Data were analysed using SAS version 9.1 (SAS Institute, Cary, NC). Unconditional logistic regression was used to estimate odds ratios (ORs), adjusted for child's age and sex.

**Ethical approval and consent to participate:** institutional Research and Ethics Committee (IREC) of Moi University and Moi Teaching and Referral Hospital approved the study. Written informed consent was obtained from parent or legal guardian of each participant.

## Results

**Figure 1:**
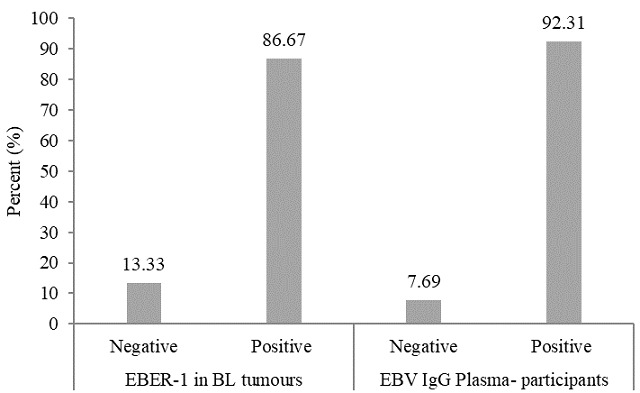
EBER-1in BL tumour and EBV IgG in participants’ sera

Ninety three (93) percent participants and 86.7% of BL cases were positive for EBV IgG antibodies in serum, and EBER-1 positive respectively, while 13.33% did not carried latent EBV encoded small RNA 1 (EBER-1) (Figure 1). All (100%) jaw BL tumours were positive for EBER-1 and 90.1% expressed MYC protein (Table 1). Bivariate associations using a log-linear model, adjusted for age and gender, showed that BL occurrence was 1.39 times greater in participants who tested positive for EBER-1 than those who tested negative, adjusted odds ratio (AOR) 1.39, 95% CI: 0.16-12.19) regardless of age and gender.

**Table 1:** Tumour site and EBER-1/MYC status in BL cases

Tumour Site	Sex M/F	EBER-1+ (%)	*MYC+*
Jaw 11(33%)	7/4	100	10/11(90.1%)
Abdomen 15(46%)	13/2	93	15/15(100%)
Jaw & Abdomen 4(12%)	3/1	100	3/4(75.0%)
Lymph Node 2(6%)	2/0	100	2/2(100%)
Thyroid 1(3%)	1/0	100	1/1(100%)

## Discussion

Nearly every participant in the study setting had been exposure to EBV as measured by EBV IgG. The association of EBER-1 and BL tumours in this current study suggest a role of EBV in BL pathogenesis in support of some past studies [8,9]. Sustained immune stimulation in response to persistent infection such as EBV is thought to trigger oncogenic mutations y during VDJH and VJL immunoglobulin genes recombination phase during B cell activation in response to a pathogen [10]. This step probably involves Ig/c-myc gene translocation as indicated by many MYC+ protein positive BL tumours in this study. The fact that not every participant was positive for EBV IgG, EBER-1 and MYC negative developed BL may be interpreted to mean that persistence of EBV is usually benign and appears to cause disease when the host-virus balance is upset [11].

## Conclusion

BL is associated with EBV in the study setting. The existence of BL tumours negative for EBER-1 and MYC Burkitt's lymphoma may be suggestive of alternative pathogenesis mechanisms or a variant(s) and needs further studies.

### What is known about this topic

- That infectious agents may play role in BL pathogenesis.

### What this study adds

- The study highlights the extent of EBV antibody and antigen in BL among children at Moi Teaching and Referral Hospital in western Kenya;
- That some BL tumours negative for EBER-1/MYC also occur in this geographic region;
- That EBER-1 negative tumours may suggest a new BL variant or alternative BL pathogenesis in this region widely known to be ecological zone for endemic BL.

## Competing interests

The authors declare no competing interests.
